# Quantification and visualization of flow in small vessels of the Circle of Willis: time-resolved three-dimensional phase contrast MRI at 7T compared with 3T

**DOI:** 10.1186/1532-429X-14-S1-W42

**Published:** 2012-02-01

**Authors:** Pim van Ooij, Jaco J Zwanenburg, Fredy Visser, Charles B Majoie, Ed vanBavel, Jeroen Hendrikse, Aart Nederveen

**Affiliations:** 1 Radiology, Academic Medical Center, Amsterdam, Netherlands; 2 Biomedical Engineering & Physics, Academic Medical Center, Amsterdam, Netherlands; 3 Radiology, University Medical Center, Utrecht, Netherlands; 4 Images Science Institute, University Medical Center, Utrecht, Netherlands

## Background

A promising technique to measure blood flow is time-resolved three-dimensional phase contrast MRI (PC-MRI). In small structures as the Circle of Willis (CoW), the resolution of the measurement needs to be high (< 1 mm^3^). Image quality may be compromised when SNR decreases with increasing resolution which leads to blood flow direction uncertainty and flow quantification inaccuracies. To increase SNR, PC-MRI can be conducted at higher field strengths. In this study time-resolved 3D PC-MRI is performed in the CoW of five volunteers at 3T and 7T to investigate the advantages of increased SNR.

## Methods

Examinations were performed on 5 healthy subjects. Retrospectively gated 3D PC-MRI was performed in an 8-channel head coil at 3T and a 16-channel head coil at the 7T MR system (both scanners Achieva, Philips Healthcare, Best, The Netherlands) at a resolution of 0.5 mm^3^. Imaging parameters: TE/TR: 4.1/8.6; flip angle: 20°; field of view: 180 x 180 x 20 mm^3^ (AP x RL x FH); velocity encoding: 150 cm/s x 150 cm/s x 150 cm/s; SENSE: 3. Background phase correction was performed by subtraction of the average phase in a static region of interest (amygdala). The lumen in both scans was semi-automatically segmented at all cardiac phases and in every slice in the fast field echo images using a level set evolution algorithm. The data was imported in GTFlow (Gyrotools, Zurich, Switzerland) to perform blood flow visualization. To allow for a voxel-wise comparison between the 7T and the 3T results, the 7T velocity information was registered and interpolated to the 3T data. Mean and standard deviation of the paired difference and the median of the angle between velocity vectors were calculated. Signal to noise was calculated in the amygdala.

## Results

**Figure 1:**
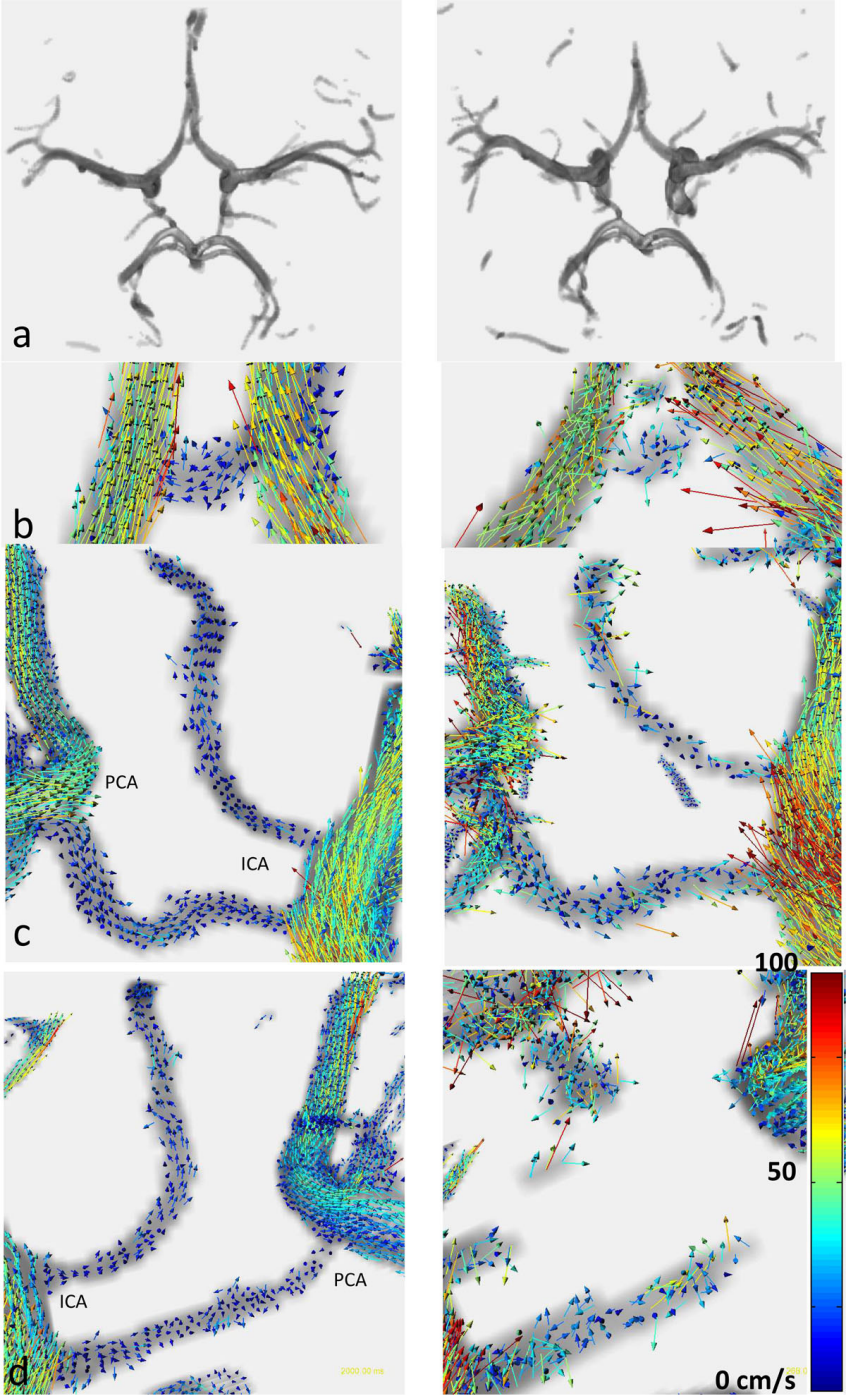
Left column: 7T, right column: 3T. Row a shows the segmented Circle of Willis. Row b displays flow in the ACoA. Row c in the left PCoA, row d in the right PCoA.

From table 1 it can be deduced that the SNR is a factor 2.7 higher in the 7T data. This gain in SNR allows for better segmentation and less noise in the velocity data, as can be seen in figure 1. At 7T it is visible that the flow through the Anterior Communicating Artery (ACoA, fig 1b) is from right to left, in the left Posterior Communicating Artery (PCoA, fig 1c) from Internal Carotid Artery (ICA) to Posterior Carotid Artery (PCA) and in the right PCoA (fig 1d) from PCA to ICA. Furthermore, note the upward flow in the anterior choroidal artery. Directions of flow are less obvious in the 3T data, due to increased noise or failure of segmentation. The noise in the 3T data results in a higher mean velocity, as shown by the positive mean of the paired differences in table 1. Standard deviations and median angles were similar for all subjects.

**Table 1:** Mean, standard deviation (SDp) of the paired difference (3T minus 7T), median angle and SNR. * indicates significant

	Vol 1		Vol 2		Vol 3		Vol 4		Vol 5	
Field Strength	3T	7T	3T	7T	3T	7T	3T	7T	3T	7T
Mean (cm/s)	0.4* (p=0)		1.2* (p=0)		0.3* (p=0)		0.2* (p=0)		0.0 (p=0.6)	
SDp (cm/s)	10.1		16.3		17.2		17.8		14.4	
Median angle (°)	21.6		27.6		26.0		35.3		31.3	
SNR	12.6	34.3	12.4	38.9	11.2	29.8	10.9	28.1	12.1	27.4

## Conclusions

Increased flow signal at 7T enhances segmentation and decreased noise allows for better visualization of flow in small vessels in the Circle of Willis.

